# Supplementary material for: DMRforPairs: identifying Differentially Methylated Regions between unique samples using array based methylation profiles
Source: BMC Bioinformatics. 2014 May 15;15:141. doi: 10.1186/1471-2105-15-141 (PMC4046028; doi:10.1186/1471-2105-15-141)
Supplement: Additional file 2 — DMRforPairs output for the comparison of A431-MCF7 and NA17018-NA17105. Please start from the HTML files in each folder. Available via the BMC Bioinformatics website. [file 1471-2105-15-141-S2.zip › 1394847754114233_MOESM2_ESM/A431_MCF7/figures/21986.pdf]

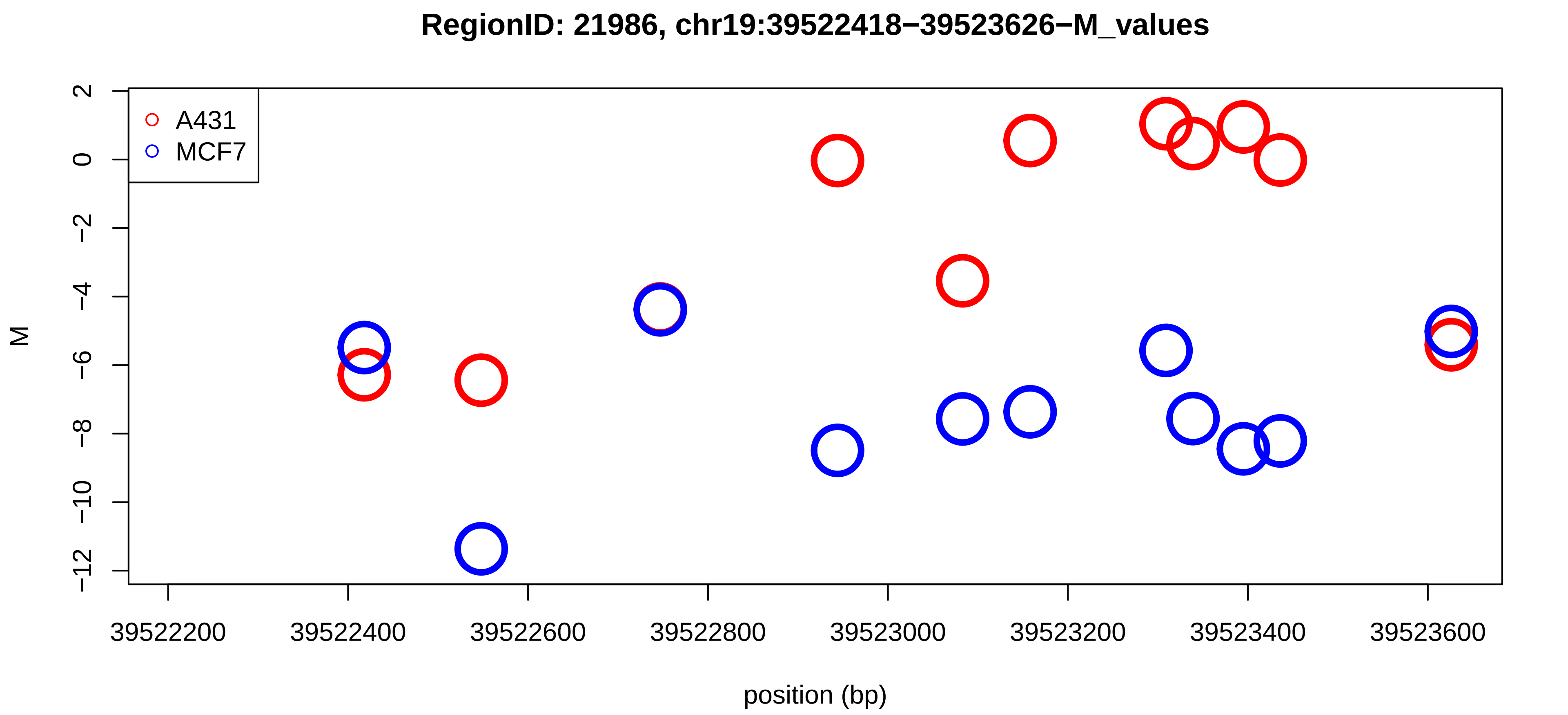
RegionID: 21986, chr19:39522418−39523626−M_values
A431
MCF7
M
2
0
−2
−4
−6
−8
−10
−12
39522200
39522400
39522600
39522800
39523000
39523200
39523400
39523600
position (bp)

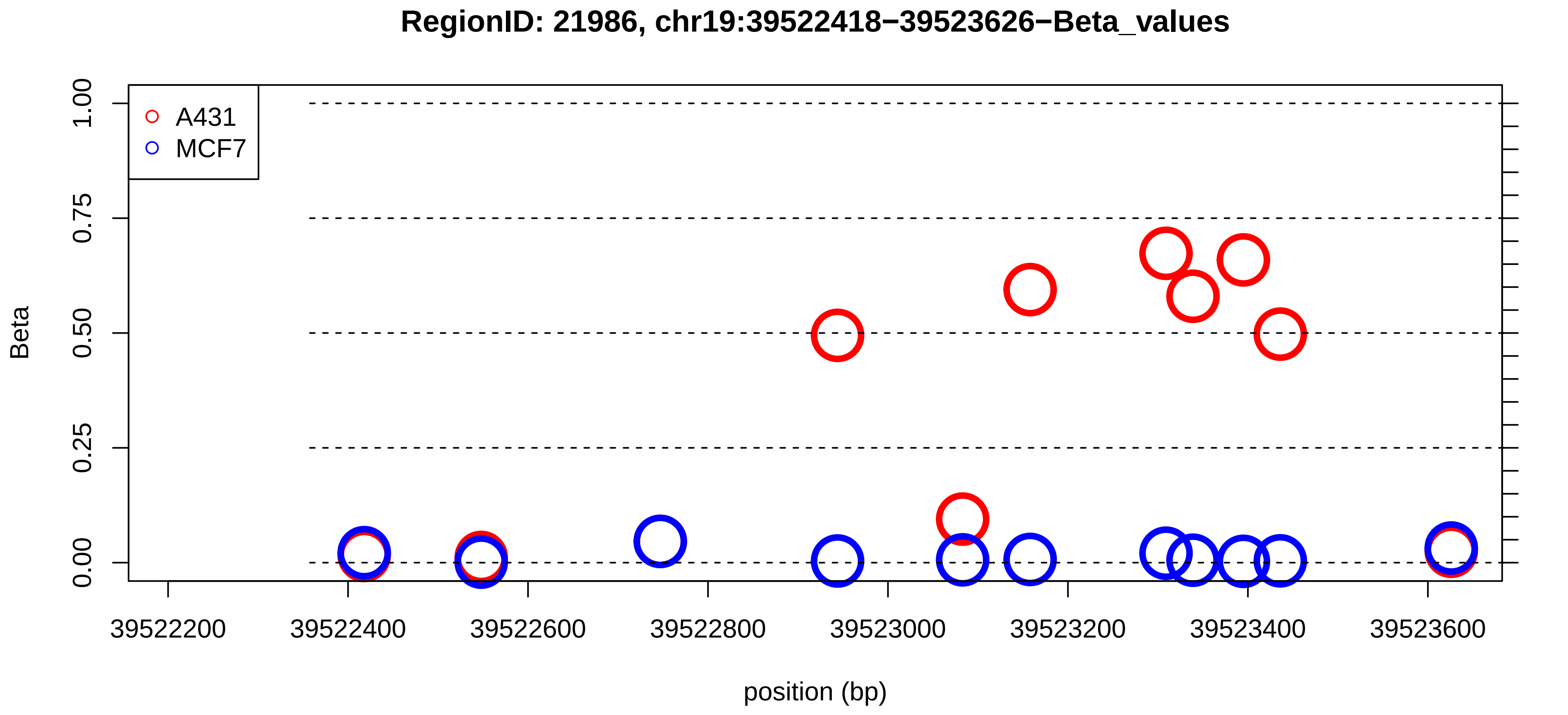
RegionID: 21986, chr19:39522418−39523626−Beta_values
A431
MCF7
Beta
1.00
0.75
0.50
0.25
0.00
39522200
39522400
39522600
39522800
39523000
39523200
39523400
39523600
position (bp)

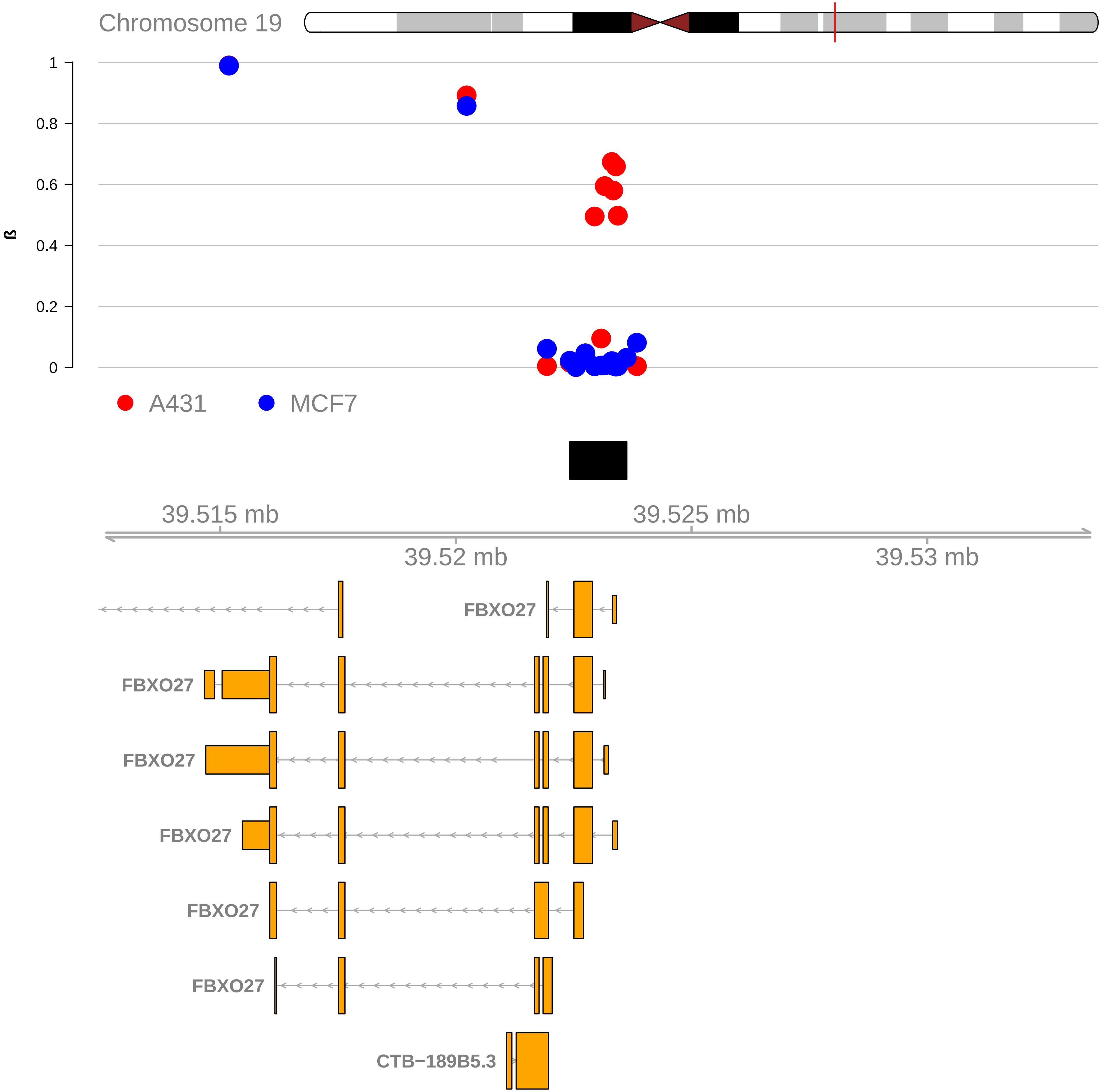

Chromosome 19
1
0.8
0.6
0.4
0.2
0
ß
A431
MCF7
39.515 mb
39.525 mb
39.52 mb
39.53 mb
FBXO27
FBXO27
FBXO27
FBXO27
FBXO27
FBXO27
CTB−189B5.3
